# The *Yersinia enterocolitica* Ysa type III secretion system is expressed during infections both in vitro and in vivo

**DOI:** 10.1002/mbo3.136

**Published:** 2013-10-24

**Authors:** Zachary W Bent, Steven S Branda, Glenn M Young

**Affiliations:** 1 Microbiology Graduate Group, University of CaliforniaDavis, CA; 2 Systems Biology, Sandia National LaboratoriesLivermore, CA; 3 Biotechnology and Bioengineering, Sandia National LaboratoriesLivermore, CA; 4 Department of Food Science and Technology, University of CaliforniaDavis, CA

**Keywords:** In vivo expression, T3SS, *Y. enterocolitica*, Ysa, Ysp

## Abstract

*Yersinia enterocolitica* biovar 1B maintains two type III secretion systems (T3SS) that are involved in pathogenesis, the plasmid encoded Ysc T3SS and the chromosomally encoded Ysa T3SS. In vitro, the Ysa T3SS has been shown to be expressed only at 26°C in a high-nutrient medium containing an exceptionally high concentration of salt – an artificial condition that provides no clear insight on the nature of signal that *Y. enterocolitica* responds to in a host. However, previous research has indicated that the Ysa system plays a role in the colonization of gastrointestinal tissues of mice. In this study, a series of Ysa promoter fusions to green fluorescent protein gene (*gfp*) were created to analyze the expression of this T3SS during infection. Using reporter strains, infections were carried out in vitro using HeLa cells and in vivo using the mouse model of yersiniosis. Expression of green fluorescent protein (GFP) was measured from the promoters of *yspP* (encoding a secreted effector protein) and *orf6* (encoding a structural component of the T3SS apparatus) in vitro and in vivo. During the infection of HeLa cells GFP intensity was measured by fluorescence microscopy, while during murine infections GFP expression in tissues was measured by flow cytometry. These approaches, combined with quantification of *yspP* mRNA transcripts by quantitative reverse transcriptase-polymerase chain reaction (qRT-PCR), demonstrate that the Ysa system is expressed in vitro in a contact-dependent manner, and is expressed in vivo during infection of mice.

## Introduction

Three species of the genus Yersinia are pathogenic to humans: *Yersinia pseudotuberculosis* and *Yersinia enterocolitica* cause gastrointestinal illnesses, while *Y. pestis* is the causative agent of bubonic and pneumonic plague. In healthy adults, *Y. enterocolitica* infections typically result in a self-limiting gastroenteritis (Cover and Aber 1989; Bottone 1999). However, in patients with compromised or undeveloped immune systems, the gastroenteritis can lead to bacteremia, with a mortality rate approaching 50% (Cover and Aber 1989). Over half of all *Y. enterocolitica* infections occur in children under the age of 5, and 38% of all infections occur in infants less than 1 year of age, the cohort that is most predisposed to developing the fatal systemic form of infection (Bottone 1999; Koehler et al. 2006). Both the gastrointestinal disease and the systemic phase of yersiniosis can be successfully recapitulated in mice, making it a particularly useful animal model for studying pathogenesis (Carter 1975).

Isolates of *Y. enterocolitica* are categorized into six distinct biovars, defined primarily on the basis of biochemical and physiological characteristics (Bottone 1999; Howard et al. 2006). Among the biovars, 1B is exceptionally pathogenic, causing the most severe illness and pathology in humans (Bottone 1999; Howard et al. 2006). Unlike the other biovars of *Y. enterocolitica*, biovar 1B is lethal to mice (Schiemann et al. 1981; Howard et al. 2006). The increased virulence of biovar 1B can be partially explained by the ∼199 kb plasticity zone, a unique region of the genome exclusive to biovar 1B. The plasticity zone includes gene clusters encoding an iron uptake system, a type IV pilus, a type II secretion system (T2SS), and the Ysa type III secretion system (T3SS). Importantly, each of these factors has been shown to contribute to virulence using the mouse model of yersiniosis (Schubert et al. 1999; Haller et al. 2000; Foultier et al. 2002; Iwobi et al. 2003; Collyn et al. 2004; Thomson et al. 2006).

*Yersinia enterocolitica* biovar 1B maintains three distinct T3SS associated with virulence: The plasmid encoded Ysc T3SS and flagellar T3SS, common to all pathogenic species of Yersinia (Cornelis 2002); and the Ysa T3SS system, which is unique to biovar 1B (Haller et al. 2000; Foultier et al. 2002; Young and Young 2002; Thomson et al. 2006). Both the Ysc and Ysa systems are required for full virulence of biovar 1B (Venecia and Young 2005). Previous examinations defined three laboratory cultivation conditions that, when combined, result in the expression of Ysa system genes and release of Ysp effectors directly into the culture medium. Growth must be in a nutrient-rich medium, the medium must contain >150 mmol/L salt (NaCl or KCl), and the cultivation temperature must be 26°C or less (Venecia and Young 2005). This combined set of conditions, not known to be physiologically relevant to any known *Y. entercolitica* host, has led some investigators to conclude that the Ysa T3SS must operate solely outside of a mammalian host (Foultier et al. 2002). Strikingly, this view appears incomplete as the Ysa system affects the outcome of gastrointestinal colonization in mice (Venecia and Young 2005). Recent work further implicated the Ysa system in having a role during infection of mammals when it was demonstrated that it targets into mammalian cells eight Ysp effectors, as well as the well-studied YopE and YopP effectors, which were originally thought to be exported only by the Ysc system (Walker and Miller 2004; Venecia and Young 2005; Matsumoto and Young 2006; Mildiner-Earley et al. 2007; Walker et al. 2010). Another recent publication revealed a role for the Ysa system during intracellular replication in Drosophila S2 cells (Walker et al. 2013), though insects are not known to carry *Y. enterocolitica*. Given these seemingly contradictory facts, it is apparent that there is a knowledge gap limiting our full understanding of the Ysa system. Therefore, the goal of this study was to gain insight on conditions and location in the mammalian host where the Ysa T3SS is expressed. We used an experimental approach that allowed for detection and measurement of Ysa gene expression during infection of cultured mammalian epithelial cells as well as in multiple tissues during the infection of mice.

## Material and Methods

### Bacterial strains and growth conditions

All bacterial strains used in this study are listed in Table S1. Overnight growth was routinely performed in 10 g/L tryptone, 5 g/L yeast extract (TYE) media at 26°C with shaking, unless otherwise noted. TYE is used throughout this study as a Ysa and Ysc T3SS non-inducing medium, except where indicated. *fliC* expression was induced by growth in TYE at 26°C. Ysa T3SS was induced using TYE supplemented with 290 mmol/L NaCl (Ysp media), with growth at 26°C (Venecia and Young 2005). Ysc T3SS was induced using Luria Broth (LB), calcium chelated with 1.5 mg/mL MgCl_2_ and 2.1 mg/mL Na_2_C_2_O_4_ (Yop media), with growth at 37°C. Ampicillin (100 μg/mL), kanamycin (50 μg/mL), naldixic acid (20 μg/mL), chloramphenicol (12.5 μg/mL), and tetracycline (7.5 μg/mL) were added to the media where indicated. HeLa cells were routinely grown in Dulbecco's Modified Eagle Medium (DMEM) (Life Technologies, Grand Island, NY) supplemented with fetal bovine serum to a final concentration of 10%. All HeLa cell growth and infections were done at 37°C in an atmosphere of 5% CO_2_.

### GFP reporter plasmids and strain construction

The entire *ysaE*-*orf6/7* region of the *Y. enterocolitica* JB580v genome (Kinder et al. 1993) was amplified by polymerase chain reaction (PCR), using the forward primer *ysaE*/*orf6*-F and the reverse primer *ysaE*/*orf6*-R (Table S2), generating a ∼950 bp fragment. This fragment was amplified using Pfu Turbo (Stratagene, La Jolla, CA) and cloned into pCR-BluntII-TOPO according to the manufacturer's instructions (Life Technologies). The promoter regions of *yspP* and *yopH* were amplified using the following primers: P*yspP*-F, P*yspP*-R, P*yopH-F*, and P*yopH*-R (Table S2); in each case this generated an ∼500 bp fragment that was then amplified and cloned into pCR-BluntII-TOPO. GFP reporter plasmids were created by subcloning either the entire gene (*ysaE*/*orf6*) or the promoter regions (*yspP* and *yopH*) from the TOPO vectors into the BamHI or XbaI site of pDW6 (Cummings et al. 2006).

Strain GY6267, which is cured of the virulence plasmid, was created by streaking strain GY5718 (Bent and Young 2010) on Yop medium agar and incubating for 2 days at 37°C. After 2 days of growth, large and small colonies were visible. The large colonies were presumed to have been cured of the plasmid, as increased growth indicated that energy was used for proliferation rather than Yop protein expression. Several large colonies were verified to be plasmid cured, by growing the strains in Yop medium at 37°C for 6 h, subjecting the conditioned medium to trichloroacetic acid (TCA) precipitation, and analyzing the precipitates by sodium dodecylsulfate polyacrylamide gel electrophoresis (SDS-PAGE) to verify the lack of secreted Yop proteins. Strains GY6397 and GY6398 were created by integrating the plasmids pGY435 and pGY526 (Walker and Miller 2004), respectively, into GY5718 through conjugation with an *Escherichia coli* S17-1 λ*pir* strain that harbored the plasmid. The *ysaE* deletion (Δ*ysaE*) strain was created by integrating the plasmid pGY518 into the chromosome of GY5718, and then growing in LB broth containing chloramphenicol and ampicillin for 6 h to enrich for strains that have lost the integrated plasmid. After isolating individual colonies on LB agar, inactivation of *ysaE* was confirmed by growing in Ysp medium for 6 h, TCA precipitation of the conditioned medium, and SDS-PAGE to verify lack of secreted Ysp proteins.

### GFP expression time courses

Strains were grown overnight in LB with ampicillin at 26°C, then subcultured to an OD_600_ of 0.1 in 100 mL of TYE with ampicillin. Growth flasks were set in a 26°C shaker at 150 rpm, and aliquots taken every hour for 11 h were centrifuged, resuspended in phosphate-buffered saline (PBS) with 0.05% NaN_3_, and stored at 4°C in the dark until analysis by flow cytometry. At the 6-h mark, an additional aliquot of each strain was subcultured to an OD_600_ of 0.1 in 100 mL of fresh TYE with ampicillin. These parallel cultures were incubated in a 37°C shaker, aliquots taken every hour for 5 h, and the aliquots processed as described above. In both cases, samples were analyzed on the flow cytometer by counting 10,000 bacteria and using the mean of the fluorescein isothiocyanate (FITC) channel as the mean GFP intensity. This experiment was performed in triplicate.

### Media induction controls

Bacteria were grown overnight in TYE media at 26°C. A quantity of 5 μL of an overnight culture was subcultured into 1 mL of either TYE or an inducing medium in a Lab-Tek®II chamber slide (Thermo Fisher, Portsmouth, NH). The inducing condition for all strains was Ysp medium with incubation at 26°C, with the exception of the *yopH*::*gfp* strains, which were induced using Yop medium with incubation at 37°C. Growth was allowed to proceed for 2 h, at which point the chamber slides were washed once with PBS, the bacteria fixed in a 0.8% paraformaldehyde solution, and the slide sealed with SlowFade®Gold with 4', 6-diamidino-2-phenylindole (DAPI) (Life Technologies). Slides were observed at 1000× magnification, and pictures taken in both phase contrast and FITC channels. Thirty individual bacteria from at least five different fields of view were analyzed for green fluorescence intensity using ImageJ software (NCBI). Background fluorescence levels were determined and subtracted out from the final intensities. The average background-subtracted fluorescence intensity from each inducing condition was compared to that from the non-inducing condition for each strain, using a Student's *t*-test to identify statistically significant differences.

### HeLa cell infections

Approximately 9 × 10^4^ HeLa cells were grown overnight in Lab-Tek®II chamber slides in 1 mL of DMEM at 37°C. Bacterial strains were grown overnight in TYE at 26°C. One microliter of overnight bacterial culture was used to infect the HeLa cells in the slide chamber, achieving an multiplicity of infection (MOI) of ∼10. Infections were allowed to proceed at 37°C for 2 h, at which point the slide chambers were washed with PBS and cells fixed in a 0.8% paraformaldehyde solution. The chambers were then washed in PBS three times, and the cells permeabilized in a solution of PBS with 3% BSA (Sigma, St. Louis, MO) and 1% saponin (Sigma). The chambers were then washed three more times with PBS, stained with Alexa Fluor® 594 phalloidin (Life Technologies), washed again, then dried. Finally, the slides were sealed with SlowFade®Gold with DAPI (Life Technologies) and allowed to sit at room temperature in the dark for at least 3 h. The slides were analyzed by fluorescence microscopy at 1000× magnification, with pictures taken in phase contrast, FITC, DAPI, and Alexa 594 channels. Green fluorescence intensity of 40–45 individual bacteria that were associated with the HeLa cells were compared to a similar number that were not associated with the HeLa cells using ImageJ software. Bacteria were determined to be associated with the HeLa cells if they were above or below the phalloidin-stained outline of the cell and appeared to be physically touching the cell. A minimum of five randomly selected fields of view from each slide were used for this analysis. Auto-fluorescence from the HeLa cells was measured in an uninfected control, and the value subtracted from the final intensities of the bacterial cells. Average intensities from the cell-associated bacteria were compared to those of non-associated bacteria, using a Student's *t*-test to identify statistically significant differences.

### Quantitative reverse transcriptase-polymerase chain reaction

Wild type and *ysaE*, *rcsB*, and *ysrS* mutant strains were grown overnight in TYE at 26°C. 50 μL of culture were transferred to each of three tubes containing 5 mL of fresh TYE, Ysp, or filter-sterilized spent DMEM, and these cultures incubated at 26°C (TYE and Ysp media) or 37°C (spent DMEM) for 2 h. Total RNA was extracted from 1 ml aliquots from each culture, using RNAzol (Molecular Research, Cincinnati, OH) followed by Direct-zol cleanup (Zymo Research, Irvine, CA), according to the manufacturers' instructions. RNA concentration was measured by Qubit (Life Technologies), and the samples diluted to a final concentration of 1 ng/μL. For infections, HeLa cells were grown to a confluent monolayer in 6-well plates, and 10 μL of an overnight culture grown in TYE at 26°C were added to the monolayer to achieve an MOI of ∼10. Bacteria were moved onto the cells by centrifugation, and the infection was allowed to proceed for 2 h at 37°C in a 5% CO_2_ incubator. All infections were performed in triplicate. After 2 h, the supernatant was removed, the cells were washed twice with PBS, and total RNA was extracted using RNAzol combined with the Direct-zol kit (Zymo Research, Irvine, CA). RNA was quantitated and diluted to a final concentration of 10 ng/μL. Primers were designed to amplify a ∼200 base pair region of the genes *yspP*, *yopH*, and *dnaK*; their sequences are listed in Table S2. 40 cycles of quantitative reverse transcriptase-polymerase chain reaction (qRT-PCR) were performed, using the iScript One-Step RT-PCR Kit (Bio-Rad, Hercules, CA) in triplicate.

### Flow cytometry

Flow cytometry analysis of the bacterial populations was performed as previously described (Bumann 2002; Cummings et al. 2006). Samples for flow cytometry were analyzed at the UC Davis Optical Biology Shared Resource on a Becton Dickenson LSRII flow cytometer using BD FACSDiva software (Becton Dickenson, Franklin Lakes, NJ). Default voltage settings were used with the exception of forward scatter, side scatter, FITC, and PE-TX-RED-A, which were optimized for use in this study. Data analysis was performed using FlowJo 7.2.4 (Tree Star, Ashland, OR). Samples were analyzed for GFP expression on the FITC channel (*x*-axis), and for background and auto-fluorescence on the PE-TX-RED-A channel (*y*-axis), using the 488 nm laser for excitation. In the FliC time course experiments, bacteria were analyzed only on the FITC channel. All samples run on the flow cytometer were re-suspended in PBS with 0.05% NaN_3_, to fix the bacteria and to preserve fluorescence (Cummings et al. 2006).

### GFP expression in vivo

Four to 6-week-old female BALB/c mice (Charles River Laboratories, Reno, NV) were used for all experiments in this study. Mice were given ampicillin at a concentration of 2 mg/mL in their drinking water for at least 3 days prior to infection and throughout the experiment, to ensure maintenance of the reporter plasmids. Bacteria were grown overnight in TYE medium at 26°C, and mice were infected with ∼10^8^ bacteria re-suspended in PBS by oral gavage. Mice were sacrificed after 48 or 120 h of infection. The terminal ileum, Peyer's patches, and mesenteric lymph nodes were harvested from the 48-h infection group; these same tissues, as well as the spleen, were harvested from the 120-h infection group. Each tissue was mechanically homogenized in PBS with 1% TritonX-100. 300 μL of each homogenate was added to 300 μL of PBS with 0.1% NaN_3_, followed by addition of 50 μL CountBright™ absolute counting beads (Life Technologies). These samples were then stored in the dark until they could be run on the flow cytometer. Flow cytometry was performed as described above, using the same voltage settings for each sample to ensure consistency across all experiments. The CountBright beads were used to ensure that for all samples a uniform volume of homogenate (12.75 μL) was analyzed. All infections were performed at least in quadruplicate, with representative data from individual mice presented in each row of Figures 4 and 6. A separate 300 μL aliquot of each homogenate was placed on ice and used for serial dilution agar plate counts, to determine the total number of bacteria present in the tissue. This number is reported in the upper left hand corner of each graph in Figures 4 and 6. Plasmid maintenance was verified by plating serial dilutions on agar plates with naldixic acid (*Y. enterocolitica*) or naldixic acid and ampicillin (*Y. enterocolitica* + plasmid).

## Results

### GFP fluorescence reflects gene expression

The in vitro and in vivo expression of bacterial virulence factors can be studied through the use of reporter strains that undergo an observable change when the gene of interest is expressed. One such method is to fuse the promoter of a virulence factor to the *gfp gene*, and use either fluorescence microscopy or flow cytometry to measure fluorescence as an indicator of gene expression (Bumann 2002; Cummings et al. 2006; Bumann and Valdivia 2007). In this study, we used the promoters of two Ysa genes (*yspP* and *orf6*) to drive expression of *gfp*, selecting them on the basis of their different roles in the T3SS as well as their chromosomal locations and differential regulation by YsaE. The *yspP* gene which encodes the effector protein YspP, is located well outside the Ysa locus, and is regulated by YsaE; the *orf6* gene encodes a structural component of the T3SS, is located within the Ysa locus, and is not regulated by YsaE (Fig. S1). The promoter of each gene, as well as 500 bases or more of upstream sequence (to include upstream regulatory elements), were cloned into the *gfp* reporter plasmids. The GFP expressed from the plasmids used in this study was shown by Cummings et al. (2006) to be very stable, with a half-life of 9.5 days when expressed by *Salmonella enterica* serovar Typhimurium. It was further shown that GFP was equally distributed to daughter cells during normal growth, and it was concluded that repression of transcription of GFP would lead to a decrease in the mean fluorescence intensity of the bacteria (Cummings et al. 2006). In order to verify these results for *Y. enterocolitica*, a time course was set up to measure decline in GFP intensity upon switching to a non-inducing condition (Fig. S2). For the purpose of this time course, it was decided to use a reporter in which the *fliC* promoter drives transcription of *gfp*. FliC, the structural subunit of the flagellum, was chosen because its expression is highly dependent on temperature and its regulation is well understood. Complete repression of *fliC* occurs at temperatures above 30°C (Kapatral and Minnich 1995; Schmiel et al. 2000). This offered the opportunity to test our reporter system in *Y. enterocolitica* using a system that is better characterized than the Ysa T3SS. Growth at 26°C was used as the *fliC*::*gfp* inducing condition, and growth at 37°C was used as a repressing condition (Kapatral and Minnich 1995; Schmiel et al. 2000).

Bacteria were grown under inducing conditions for 6 h, a point at which it was experimentally determined that GFP intensity was rapidly increasing. In this experiment, bacterial cultures were analyzed by flow cytometry to determine the average fluorescence intensity of ∼10,000 bacteria at each time point. At the 6-h time point, bacterial populations were shifted to the repressing condition. The negative control in this and subsequent experiments was plasmid pDW6 (Cummings et al. 2006), which carries a promoterless *gfp*. In the *fliC*::*gfp* strain, GFP intensity quickly decreased to background levels upon switching to the repressing condition, thus demonstrating that a drop in GFP intensity reflected a decrease in gene expression from the promoter of interest (Fig. S2). In contrast, bacteria maintained under the original inducing conditions continued to display an increase in GFP intensity. The negative control strain had negligible levels of GFP fluorescence throughout the 11-h experiment, regardless of growth conditions. This demonstrates that switching to *fliC*-repressing conditions led to an immediate and permanent decrease in mean GFP intensity, indicating that GFP intensity and gene expression are correlated. Given these data, it was determined that the level of fluorescence from a bacterium expressing GFP from a regulated promoter could be used as an accurate indication of gene expression from the promoter of interest.

### GFP reporters behave as expected in media controls

To ensure that the *ysa*::*gfp* fusions functioned in a manner consistent with previous work (Venecia and Young 2005), the mean GFP intensity was determined for bacterial reporter strains grown for 2 h in conditions known to either repress or induce the Ysa system. We chose to analyze the Ysa system by observing expression of strains carrying either *orf6*::*gfp* or *yspP*::*gfp* fusions described in the previous section. As a positive control in this and subsequent experiments, plasmid pDW5 (Cummings et al. 2006) was used. Plasmid pDW5 encodes the promoter of the constitutively expressed *tetA* gene transcriptionally fused to *gfp*. Strains containing the pDW5 and pDW6 plasmids were used as positive and negative controls, respectively. These control strains were grown in either TYE medium (non inducing) or Ysp medium (inducing). A third control, a *yopH*::*gfp* fusion, was constructed to ensure that *gfp* expression could be reliably driven from a *Y. enterocolitica* virulence factor promoter with known and well characterized regulation (Straley et al. 1993). Additionally, the Ysa reporters (*orf6*::*gfp* and *yspP*::*gfp*) were placed into *ysaE*, *rcsB*, and *ysrS* mutant strains to verify that GFP expression from the Ysa promoters was regulated as expected in vitro by the previously described regulators YsaE, RcsB and YsrS (Walker and Miller 2004). Previous studies have shown that the two-component regulators YsrS/R and RcsC/B positively regulate expression of the Ysa T3SS, and that YsaE regulates structural components of the system but not secreted effectors (Walker and Miller 2004, 2009; Venecia and Young 2005; Walker et al. 2010). Similarly, as a control for the *yopH*::*gfp* expression, a pYV (Yersinia virulence plasmid) cured strain was used to render it defective for its key positive regulator VirF. GFP intensity of the bacteria was measured by fluorescence microscopy.

**Figure 1:**
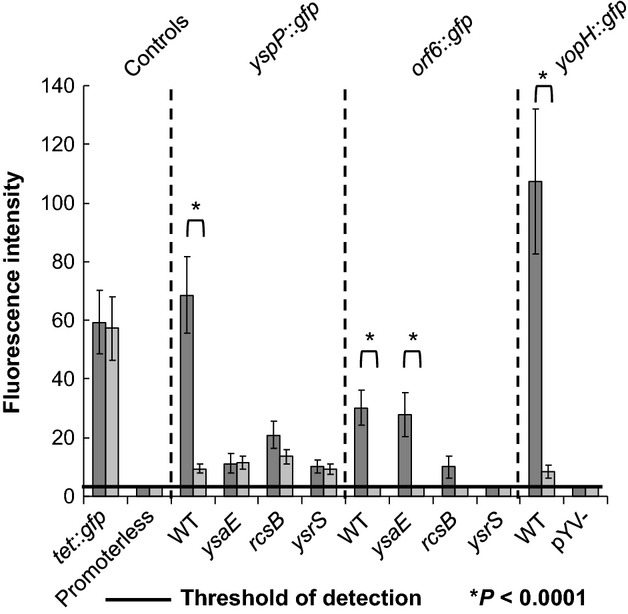
Media induction controls. A wild type and *ysaE*, *rcsB*, *ysrS*, and pYV-regulatory mutant strains containing the indicated reporter plasmids were grown for 2 h under conditions known to induce (Ysp media at 26°C for the Ysa T3SS; Yop medium at 37°C for the Ysc T3SS) or repress (TYE medium at 26°C) T3SS expression. Fluorescence intensity was measured by microscopy using ImageJ software. Dark gray bars represent bacteria that were grown in the inducing condition, and light gray bars represent those bacteria grown in the non-inducing condition. The threshold of detection corresponds to the point at which bacteria could no longer be distinguished from background fluorescence. Asterisks indicate statistically significant differences in gene expression levels between the inducing and non-inducing conditions.

The results of this analysis showed that when constitutively expressed from the *tetA* promoter, the level of GFP intensity is not affected by different growth conditions (Fig 1). However, as expected, expression from the *yspP* promoter requires proper inducing conditions as well as the *ysaE*, *rcsB*, and *ysrS* genes. For the *orf6* promoter, GFP expression required inducing conditions as well as *ysrS* and *rscB*, but not *ysaE*. We tested the effect of the *yopH* promoter on GFP expression as well. In this case, expression occurred under the Ysc-inducing condition and required the presence of *virF*. These results indicate that all reporter strains and controls functioned as expected, and that the mean GFP intensity from the reporter strains reflected Ysa expression. Furthermore, the regulation of the Ysa system by YsaE, RcsB, and YsrS occurred as previously described (Walker and Miller 2004).

### The Ysa system is expressed in a contact-dependent manner at 37°C during HeLa cell infection

**Figure 2:**
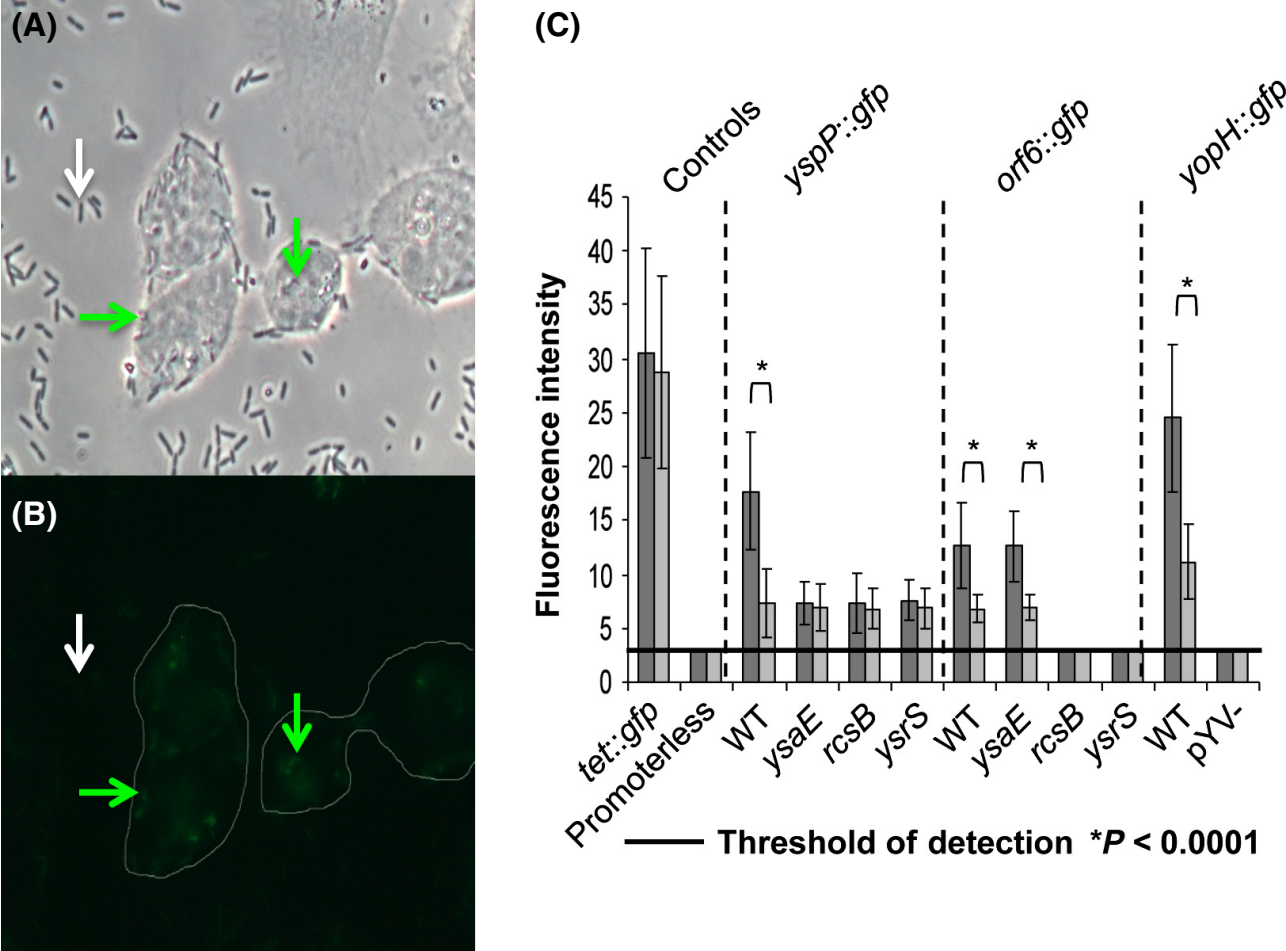
HeLa cell infections. HeLa cells were infected for 2 h with the indicated bacterial strains. Cultures were then fixed, stained, and examined by fluorescence microscopy using ImageJ software. (A) Bright field view of HeLa cells infected with the WT *yspP*::*gfp* strain, showing bacteria that are associated or not associated with the cells. (B) Alexa 488 fluorescent view of the same image with an outline of the HeLa cells drawn in. Notice that bacteria associated with the cells have higher fluorescent intensity. White arrows indicate bacteria that are not in contact with the HeLa cells and are thus are not in the Alexa 488 view. Green arrows indicate bacteria that are in contact with the HeLa cells; note that they express *gfp*. (C) Dark gray bars represent fluorescence intensity of those bacteria that were determined to be in direct contact with the HeLa cells, whereas the light gray bars correspond to those bacteria that were not associated with the HeLa cells.

Cultured epithelial cells have been used to model various aspects of bacterial infection, including cell surface binding and cellular invasion. Given that *Y. enterocolitica* is known to colonize and infect human epithelial cells (Devenish and Schiemann 1981), we examined HeLa cells infected with the GFP reporter strains at 37°C as a generic human epithelial cell model of infection. When infected HeLa cells were viewed by fluorescence microscopy, it was noted that bacteria that were in contact with the HeLa cells fluoresced with more intensity than those bacteria in the same field of view but not in contact with the HeLa cells (Fig. 2A, B). Because T3SS are typically said to be contact-dependent (Galan 1996; Mounier et al. 1997), this result fits well with expectations. However, this is the first time that the Ysa system has been shown to be expressed at 37°C or in a contact-dependent manner.

Strikingly, when the levels of fluorescence intensity were quantified, by averaging the fluorescence of at least 40 individual bacteria using ImageJ software to measure the intensity, it was demonstrated that the difference between HeLa cell-associated and non-associated bacterial populations was statistically significant (*P* < 0.0001) (Fig. 2C). This was true for only the reporter strains with a wild-type background and for the *orf6*::*gfp*, *ysaE* mutant strains. As mentioned above, YsaE does not regulate the transcription of proteins that make up the Ysa T3SS apparatus. Expression of *yspP*::*gfp* was dependent on *ysaE*, *rcsB*, and *ysrS*, and expression of *orf6* was dependent on *rcsB* and *ysrS*. Importantly, these data demonstrate not only that the Ysa system is expressed in a contact-dependent manner using a model that mimics bacterial interactions during a mammalian infection but, also that this expression is regulated by the known Ysa regulators.

### qRT-PCR quantification of Ysa expression in vitro

**Figure 3:**
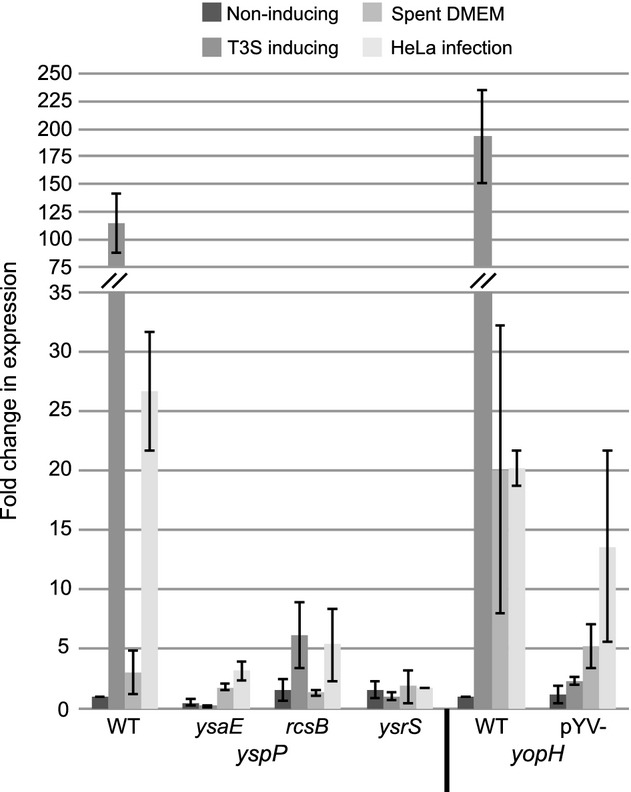
qRT-PCR analysis of *yspP* and *yopH* expression. Bacteria were grown for 2 h under conditions that do not induce expression of either T3SS (non-inducing) or induce expression of T3S (sterilized spent DMEM, infection of HeLa cells). Expression of the T3SS effector encoding genes *yspP* and *yopH* was compared in each mutant and condition to the wild type strain grown under non-inducing conditions, using *dnaK* as normalization standard. Note the ∼27-fold increase in *yspP* expression in the wild-type strain during HeLa cell infection.

As a separate, nonreporter-based means of measuring expression of the Ysa system in pure culture and its contact dependency during tissue culture infection, qRT-PCR was performed to measure mRNA levels of the genes encoding the T3SS effectors YspP and YopH. The YspP effector was chosen because it was shown in this study (Fig. 1), as well as by Matsumoto and Young (2006), to be highly expressed under Ysa inducing conditions. As controls, bacteria were grown for 2 h in the T3SS inducing conditions (Ysp medium at 26°C or Yop medium at 37°C) or non-inducing conditions (TYE at 26°C), as well as in filter-sterilized spent DMEM at 37°C. Filter-sterilized spent DMEM was used to determine if expression of the T3SS effectors was dependent on host cell contact or induced by other secreted host cell factors. In each growth condition and in the HeLa cell infection, expression of *yspP* was measured in the wild type and *ysaE*, *rcsB*, and *ysrS* mutant strains, and compared to the wild-type strain grown in the non-inducing condition, using the housekeeping gene *dnaK* as a normalization standard (Livak and Schmittgen 2001). Expression of *yopH* in both the wild type and virulence plasmid cured strains was measured in the same way. Figure 3 presents the fold change in expression of both effectors under each growth condition and in each mutant background, in comparison to wild-type growth under non-inducing conditions.

Consistent with earlier results (Figs. 1 and 2), the wild-type strain exhibited high levels of *yspP* and *yopH* expression when grown under T3SS inducing conditions and during infection of HeLa cells. Interestingly, there was no significant difference in *yspP* expression between the non-inducing condition and the sterile spent DMEM at 37°C, suggesting that it was contact with the HeLa cells, rather than secreted host factors such as cytokines, that induced expression of *yspP* during infection. The mutant Ysa regulator strains (*ysaE*, *rcsB*, *ysrS*) behaved as expected, exhibiting insignificant *yspP* expression regardless of growth condition. When combined with the results shown in Figure 2, these data provide clear indication that the Ysa T3SS is expressed in a contact dependent manner during infection of HeLa cells. In contrast to *yspP*, *yopH* appears to have similar expression levels in both spent DMEM and during HeLa cell infection, which would appear to contradict the results shown in Figure 2. It should be noted, however, that expression of the Ysc T3SS has been shown to be induced at 37°C, due to changes in the secondary structure of the virulence plasmid (Rohde et al. 1999) and the ribosome-binding site of the transcriptional activator LcrF (Bohme et al. 2012) at that temperature.

### The Ysa system is expressed in vivo throughout the course of infection

**Figure 4:**
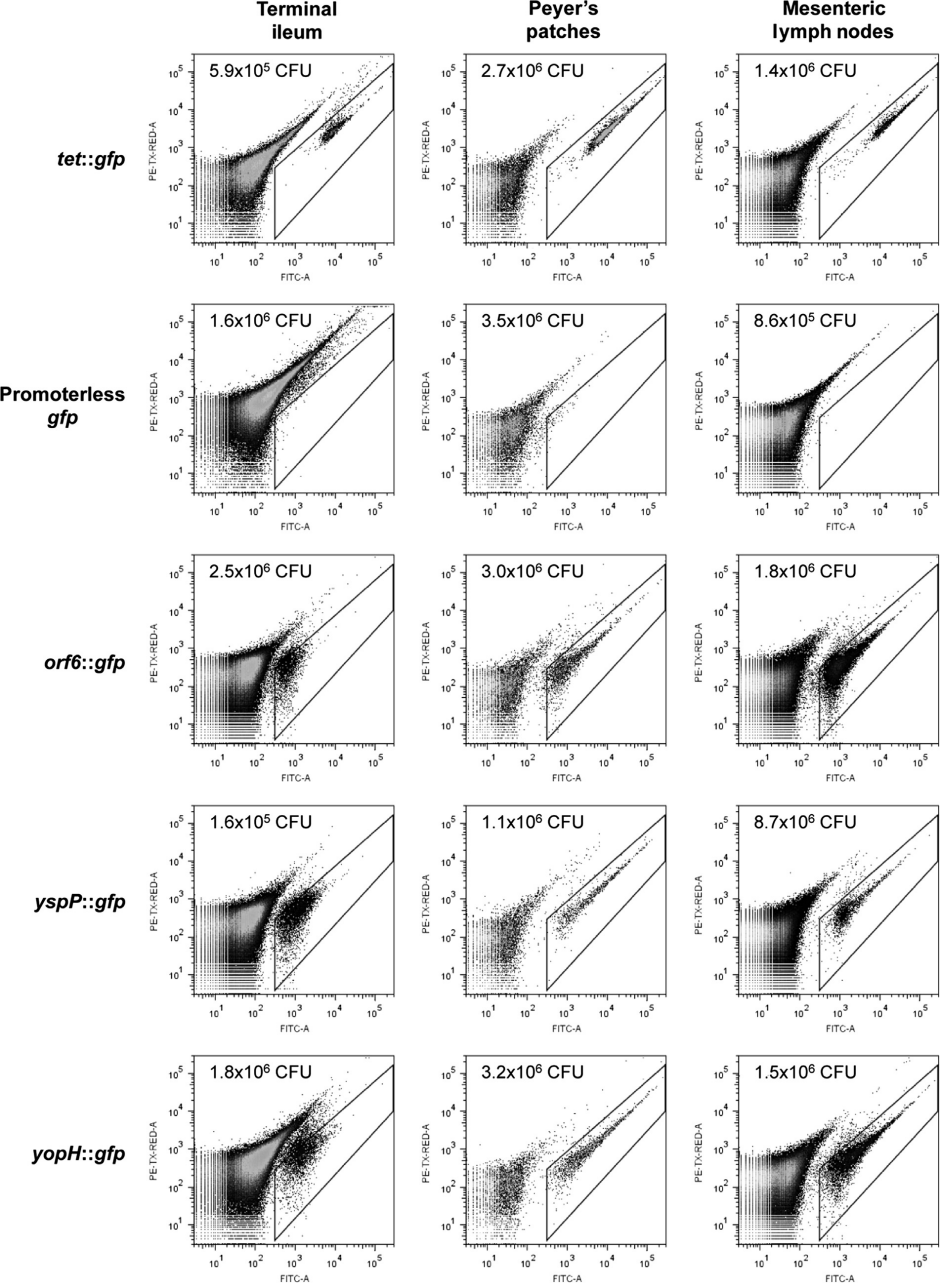
Analysis of 48-h infections by flow cytometry. 48 h post infection tissues were harvested, homogenized, and analyzed by flow cytometry. The PE-TX-RED-A axis (*y-*axis) depicts the auto-fluorescence of the mouse tissues, whereas the FITC-A axis (*x-*axis) depicts the GFP intensity of the bacterial population. Events in the gated region represent bacteria that are expressing GFP. The number in the upper left corner of each graph is the number of CFU/tissue as determined by a direct plate count of the tissue homogenates. Notice, there are large populations of bacteria in the gated areas from both Ysa T3SS promoters in all three gastrointestinal tissues.

**Figure 5:**
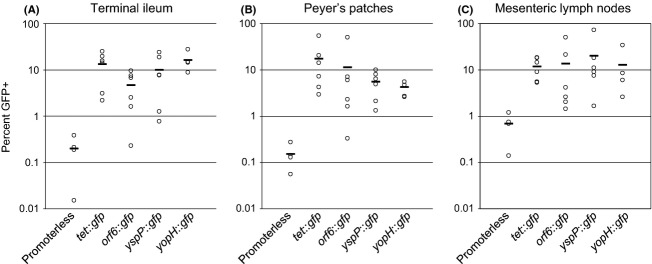
Quantification of 48-h infection results. To objectively analyze the data generated by flow cytometry, the number of bacteria in the gated region (GFP+) was compared to the total number of bacteria present in the samples based on plate counts (Total) to calculate the percentage of GFP+ bacteria. Open circles represent the results from a single mouse, in the tissue indicated; the bar depicts the average of all mice. Each strain was compared to the promoterless control, using the Mann–Whitney *U*-test. All results were found to be statistically significant, with *P-*values of ≤0.02. (A) Terminal ileum (B) Peyer's patches (C) Mesenteric lymph nodes.

**Figure 6:**
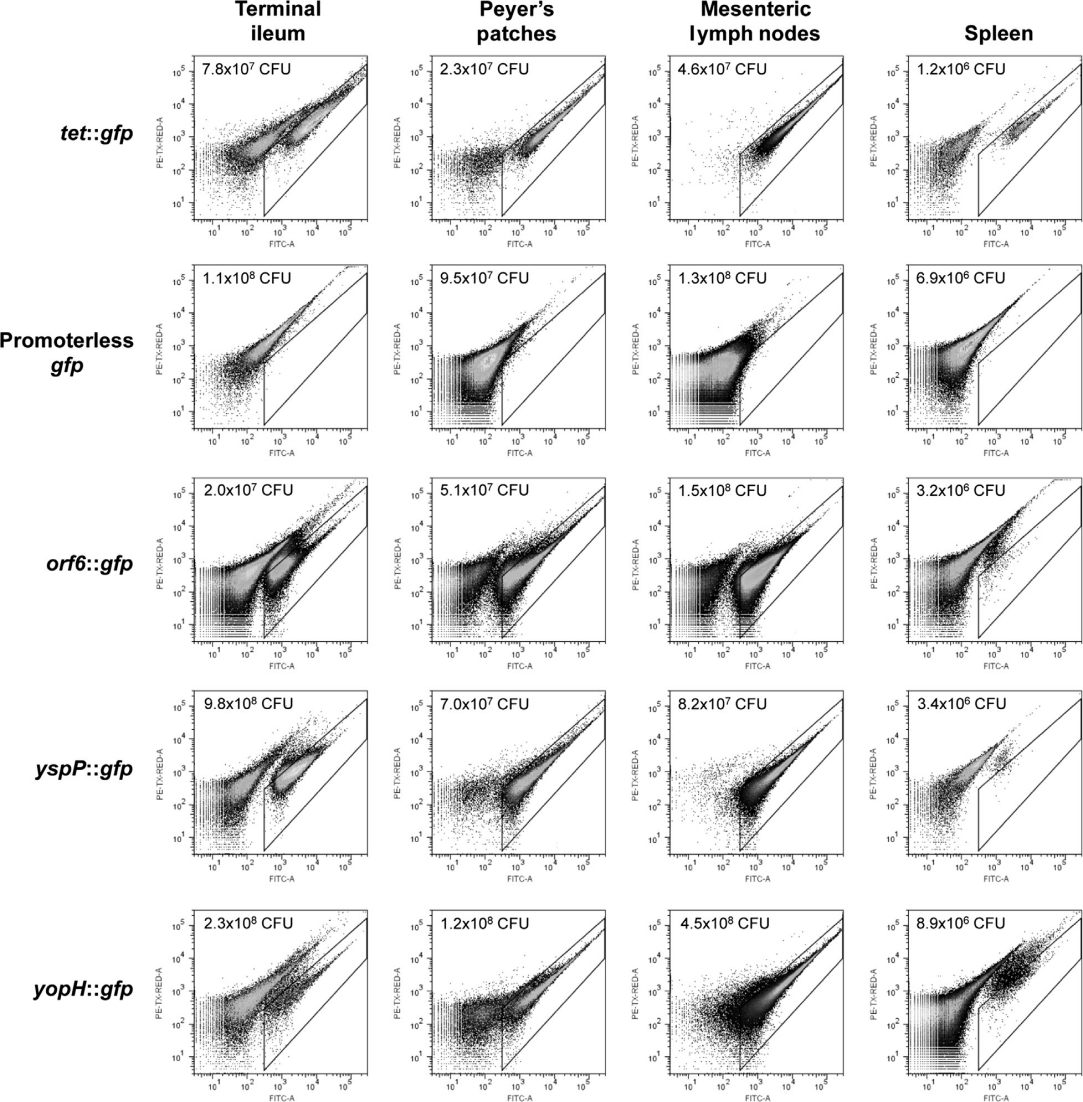
Analysis of 120-h infections by flow cytometry. Samples were analyzed identically to the samples from the 48-h infections (Fig. 4), except for the addition of the spleen as a representative systemic organ.

**Figure 7:**
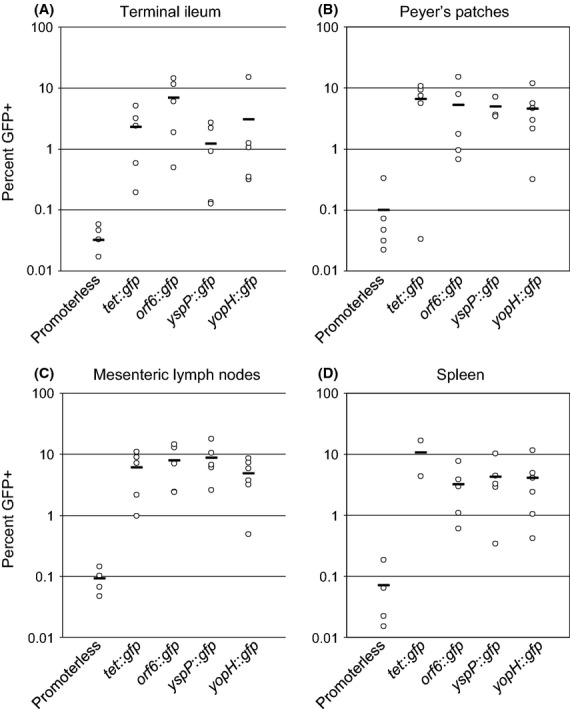
Quantification of 120-h infection. Data from the 120-h infections were treated identically to those from the 48-h infections (Fig. 5). Open circles represent the results from a single mouse, in the tissue indicated; the bar depicts the average of all mice. All results were statistically significant, with *P-*values of ≤0.02. (A) Terminal ileum (B) Peyer's patches (C) Mesenteric lymph nodes (D) Spleen.

Given the previous results indicating that the Ysa T3SS functions in vitro in a contact-dependent manner during infection of human epithelial cells, it was necessary to determine if the Ysa system is also expressed in vivo. To establish the tissues and time points where the Ysa system is expressed, mice were infected with each reporter strain as well as the positive and negative controls. The infections were allowed to proceed for either 48 (Figs. 4 and 5) or 120 h (Figs. 6 and 7). Tissue homogenates were analyzed by two-color flow cytometry, in order to distinguish bacterial populations expressing GFP from tissue auto-fluorescence. For the 48-h infections, only gastrointestinal tissues were examined, as not enough time had elapsed for large numbers of bacteria to exit the gut and begin the systemic phase of infection (Carter 1975). By 120 h, the infection had become systemic and all mice were moribund. For these mice, the gastrointestinal tissues were sampled and, in addition, the spleen was examined as a representative systemic tissue.

The graphs in Figure 4 represent single tissue homogenates analyzed by flow cytometry for auto-fluorescence from the mouse tissues (*y-*axis) and green fluorescence from GFP-expressing bacteria (*x*-axis). Each row of graphs in Figure 4 corresponds to results from a single, representative mouse. The gated area in each graph contains the bacterial population determined to be expressing GFP above the auto-fluorescence threshold (GFP+). This gate was universally applied to all samples in Figure 4, as well as to the remaining samples in the study. The values posted in the upper left corner of each graph are the number of colony forming units (CFUs) of all plasmid maintaining *Y. enterocolitica* recovered by agar plate counts from the tissue. In designing this study, it was vital to make certain that the *Y. enterocolitica* recovered from the mice maintained the reporter plasmid. If bacteria lost the plasmid during the course of infection they would be found in the population not expressing GFP whether or not the Ysa system was being expressed, thus invalidating the results. To verify that the recovered bacteria maintained the GFP reporter plasmid, all agar plate counts were done on plates containing ampicillin. This number was compared to the total *Y. enterocolitica* population found by parallel plating on agar plates without ampicillin. The numbers of CFUs from both sets of plates were equal in all tissues after both 48 and 120 h of infection (data not shown), indicating that the plasmid is effectively maintained throughout the course of the infection, and thus that the results of gene expression in vivo are valid.

In Figure 4 it is shown that there are bacterial populations expressing GFP within the gated area in both of the *orf6*::*gfp* and *yspP*::*gfp* fusions in all of the tissues examined. The *yopH*::*gfp* control also has large bacterial populations within the gated region, as would be expected. This indicates that the Ysa system is expressed during a mammalian infection in the terminal ileum, Peyer's patches, and mesenteric lymph nodes. In the spleen, after 120 h of infection, there are also many bacteria that fall within the gated region, indicating expression occurs during the systemic phase of the disease as well (Fig. 6). These results demonstrate, at least qualitatively, that the Ysa system is actively expressed during the entire course of an infection.

To quantitatively analyze the flow cytometry data, it was necessary to find a way to normalize the data. This was achieved by counting the number of bacteria in the gated region of each graph (GFP+) and comparing it to the total number of bacteria that should have been present in the sample based on plate counts, to calculate the percentage of bacteria expressing GFP. The amount of homogenate analyzed by the flow cytometer was kept consistent by using CountBright beads. These data are presented in Figures 5 and 7. Each open circle represents the results from a single mouse, in the tissue indicated, with the bar depicting the mean of all mice. The positive controls, as well as the *orf6*, *yspP*, and *yopH* reporters, were compared to the promoterless control using the Mann–Whitney *U*-test. In each case, the difference between the negative control and promoters of interest was an order of magnitude or higher. Furthermore, this difference was statistically significant in all cases, with a *P*-value of 0.02 or less. Combining these quantitative results with the qualitative results from Figures 4 and 6, it is clear that the Ysa T3SS is expressed throughout the course of an infection in a mammalian host. This is the first definitive evidence that the Ysa system is expressed during oral infection of a susceptible animal. It is also important to note that, contrary to our original hypothesis that expression would occur only in the initial stages of infection and predominantly in the gastrointestinal tissues, we found that expression occurs throughout the course of infection and in all tissues analyzed.

## Discussion

To analyze expression of the Ysa T3SS system in vivo, we chose to use a GFP reporter system in which the promoter and upstream region of the *yspP* and *orf6* genes were used to drive expression of promoterless *gfp*. Cummings and colleagues successfully used this system to study in vivo expression of several genes in *Salmonella enterica* Serovar Typhimurium (Cummings et al. 2006). During *Y. enterocolitica* infection, bacteria first colonize the gastrointestinal tract and then are quickly taken up by the Peyer's patches, where they continue to replicate at a rapid rate (Cover and Aber 1989). As with all species of Yerisina, *Y. enterocolitica* demonstrates tropism for lymphatic tissues, and therefore migrates through the lymphatic system from the Peyer's patches to the mesenteric lymph nodes, the draining lymph nodes of the gastrointestinal tract (Balada-Llasat and Mecsas 2006). There the bacteria grow until they are able to escape the lymph nodes and begin the systemic phase of infection. In all of the tissues examined in this study, the bacteria counts are increasing by as much as 10-fold per day. This proliferation effectively dilutes out the stable GFP protein that is expressed from the Ysa promoters used in this study, ensuring that only the bacteria that are actively expressing *gfp* from the Ysa promoters show green fluorescence above background levels in fluorescence microscopy and flow cytometry analyses.

Previous in vitro studies of the Ysa system revealed that expression is induced during growth at 26°C and 290 mmol/L NaCl, a condition which would seem to preclude expression in a mammalian host (Haller et al. 2000; Venecia and Young 2005). However, work in our lab suggested that there was an in vivo phenotype for a Ysa T3SS mutant during the earliest stages of mammalian infection (Venecia and Young 2005). These seemingly contradictory findings suggested that our understanding of the Ysa T3SS was incomplete. The purpose of this study was to challenge the notion that Ysa T3SS expression does not occur under conditions physiologically relevant to a mammalian host. It was also anticipated that this study would identify specific tissues and time points in which expression of the Ysa system could be demonstrated, providing insight as to how the Ysa system might influence the outcome of a mammalian infection.

Given that the Ysa T3SS had been previously examined only at 26°C in a high salt media, studies had not been done to examine expression during the infection of tissue culture cells in their normal growth media and temperature. While it is generally accepted that T3SS are contact-dependent (Galan 1996; Mounier et al. 1997), this had not been conclusively demonstrated for the Ysa T3SS. This study confirms that the Ysa system functions in a contact-dependent manner during infection of human epithelial cells. Additionally, this expression was regulated by the same proteins known to regulate the Ysa system at 26°C in the high salt media. These results demonstrate a contact-dependent phenotype under conditions that mimic those within a mammalian host. This new information led the project to move from the in vitro tissue culture model to the mouse model of yersiniosis, a model in which it would be possible to definitively test our hypothesis that the Ysa T3SS is expressed during the course of an infection in a mammalian host.

Previous work using the mouse model of yersiniosis determined that at 48 h post infection, the gastrointestinal tissues would be well colonized by *Y. enterocolitica* but the bacteria would not have exited the gastrointestinal tract to begin the systemic phase of infection (Carter 1975). An infection length of 120 h ensured that the bacteria would have had sufficient opportunity to migrate from the gastrointestinal tissues, leading to high numbers of bacteria in systemic organs. To be consistent with previous studies, the gastrointestinal tissues chosen for analysis were the terminal ileum, the Peyer's patches, and the mesenteric lymph nodes (Haller et al. 2000; Venecia and Young 2005; Matsumoto and Young 2006). It was determined that the spleen would make the best representative organ for the systemic infection, as high numbers of bacteria could be recovered and, unlike the liver, the spleen homogenates did not have eukaryotic cells vastly outnumbering bacterial cells.

After 48 h of infection, we found that there were significant populations of bacteria expressing GFP from the Ysa promoters in each of the gastrointestinal tissues. These populations demonstrated that the Ysa T3SS is indeed expressed in vivo, and that the expression profile is similar to that of the Ysc T3SS in the *yopH*::*gfp* strain. The expression profiles for the Ysa and Ysc systems were similar after 120 h of infection. Expression of GFP was seen from the Ysa promoters in all of the gastrointestinal tissues as well as in the spleen. The fact that there is such a high level of expression of the Ysa T3SS in all the tested tissues helps to explain the phenotype of decreased competitiveness seen previously in Ysa T3SS mutants in the gastrointestinal tissues (Venecia and Young 2005).

This study dispels the idea that the Ysa T3SS is not expressed during a mammalian infection. It is clearly demonstrated that not only is the Ysa T3SS expressed during the course of an infection, but that this expression is not limited to the gastrointestinal tissues. Combined with previous work in our lab (Matsumoto and Young 2006), this suggests a clear role for the Ysa system in colonization of the gut and the subsequent systemic phase of the disease. It seems likely that the Ysa T3SS is maintained by *Y. enterocolitica* biovar 1B because it provides an advantage during the rapid colonization of the gastrointestinal tract, and perhaps in colonization of the major systemic organs as well. This rapid colonization of the gut may help to partially explain the increased pathogenicity of biovar 1B over less virulent strains. Bacteria that are able to quickly and efficiently colonize the gut may be able to spread systemically before a large-scale immune response can be mounted. With an in vivo role for the Ysa T3SS established, it will be important to identify a medium or in vitro system in which Ysa expression at 37°C can be modeled. Future research will be necessary to determine the nature of Ysa T3SS expression at 37°C, and the relevance of the current 26°C-high salt model of expression.

## Supporting Information

**Figure S1.** The organization of the Ysa locus. The Ysa locus is located within the plasticity zone, a chromosomal region unique to *Y. enterocolitica* biovar 1B, which encodes several virulence factors. The locus consists of 29 genes with the indicated functions. Note that *yspP*, a gene encoding a secreted effector of the Ysa T3SS, is located outside of the Ysa locus.

**Figure S2.** GFP intensity reflects *gfp* expression. Bacteria were cultured under a *fliC* inducing temperature (26°C) and allowed to grow for 11 h, with samples being taken every hour for analysis of mean GFP intensity. After 6 h of growth, an aliquot was subcultured into a non-inducing condition (37°C). (A). The *fliC*::*gfp* reporter strain exhibits an immediate and permanent drop in mean GFP intensity upon switching to the non-inducing condition. Note that plasmid pSRB1, the *fliC*::*gfp* reporter used in this study, carries the *fliC* promoter of *Salmonella enterica* Serovar Typhimurium. (B). The promoterless *gfp* reporter serves as a negative control, with negligible GFP expression regardless of growth condition. Note the *y*-axis scale difference between panels (A and B).

**Table S1.** Bacterial strains and plasmids used in this study.

**Table S2.** Primers used in this study.
