# Supplementary figures and images for: The Yersinia enterocolitica Ysa type III secretion system is expressed during infections both in vitro and in vivo
Source: Microbiologyopen. 2013 Oct 24;2(6):962–75. doi: 10.1002/mbo3.136 (PMC3892342; doi:10.1002/mbo3.136)

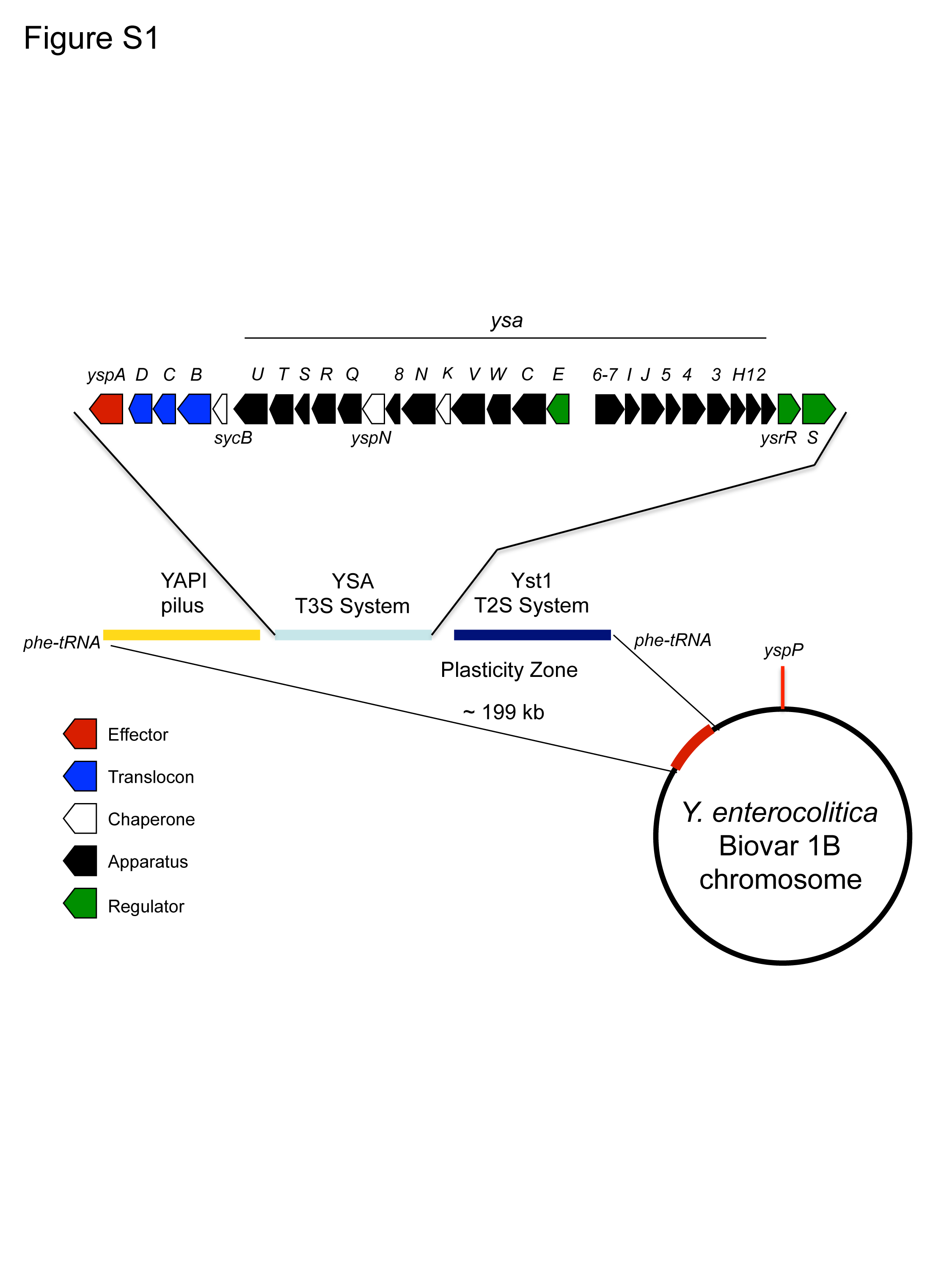

Supplement: Supplementary file 1 [file mbo30002-0962-SD1.tif]

Figure S2

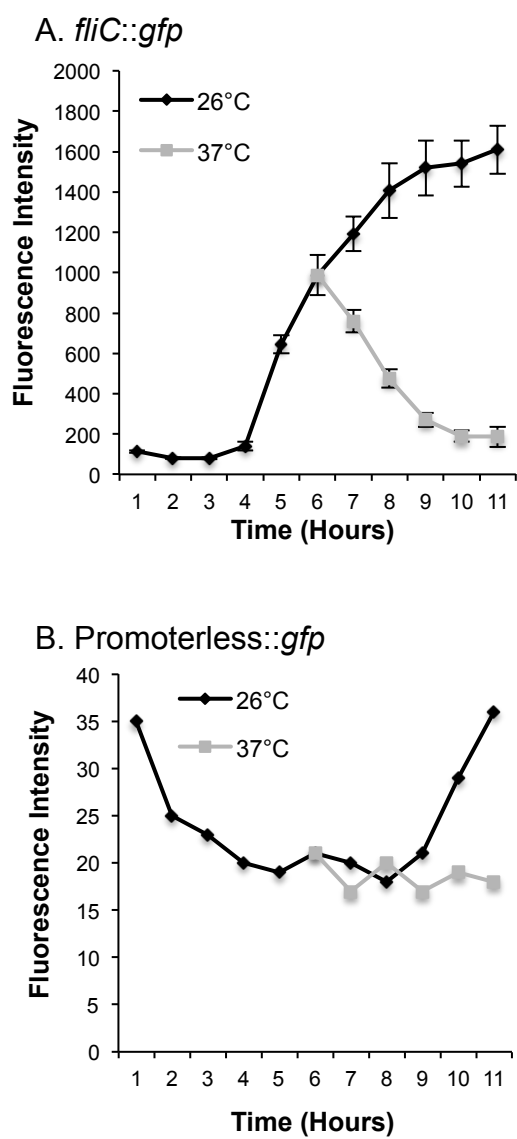

Supplement: Supplementary file 4 [file mbo30002-0962-SD4.pdf]
