# Supplementary material for: The Yersinia enterocolitica Ysa type III secretion system is expressed during infections both in vitro and in vivo
Source: Microbiologyopen. 2013 Oct 24;2(6):962–75. doi: 10.1002/mbo3.136 (PMC3892342; doi:10.1002/mbo3.136)
Supplement: Supplementary file 5 [file mbo30002-0962-SD5.pdf]

Supplementary Table 1: Bacterial strains and plasmids used in this study

Strain or Plasmid	Description	Reference
Y. enterocolitica		
JB580v	Serogroup O:8, Nal, Δ*yenR* (R- M+)	Kinder et al., 1993
GY5718	JB580v Δ*blaA*, Δ*blaB*	Bent and Young, 2010
GY6267	GY5718 pYV-	This Study
GY6397	GY5718 *rcsB*::pEP185.2	This Study
GY6398	GY5718 *ysrS*::pEP185.2	This Study
GY6399	GY5718 Δ*ysaE*	This Study
E. coli		This Study
DH5α	F- φ80d*lacZ*M15 (*lacZYA-argF*)U169 *deoR recA1 endA1*	Life Technologies
S17-1 λ*pir*	*recA thi pro hsdR*- *hsdM*+ RP4::2-Tc::Mu::Km Tn7 λ*pir*	Miller and Mekalanos, 1988
**Plasmids**		
pSRB1	p*fliC*::*gfp*	Cummings et al., 2006
pDW5	p*tetA*::*gfp*	Cummings et al., 2006
pDW6	Promoterless *gfp*	Cummings et al., 2006
pGY435	Ca. 0.5-kb fragment of *rcsB* in pEP185.2	Venecia and Young, 2005
pGY518	pEP185.2 Δ*ysaE*	B. Young and G. Young, Unpublished
pGY526	Ca. 0.5-kb fragment of *ysrS* in pEP185.2	Venecia and Young, 2005
pGY996	pDW6::*orf6*	This Study
pGY1045	pDW6::*yspP*	This Study
pGY1049	pDW6::*yopH*	This Study

Supplementary Table 2: Primers used in this study

**Primer**	**Sequence**
**Cloning**	
ysaE/orf6-F	5’ – AGATCTGTAATCACCTCAGCGTTG – 3’
ysaE/orf6-R	5’ – AGATCTTATTGACCAGCGACTTGC – 3’
P*yspP*-F	5’ – GGATCCCGCAATAGCATTTGCCAC – 3’
P*yspP*-R	5’ – TCTAGACATTTCAGGTGTTGGGGC – 3’
P*yopH*-F	5’ – ACATCTCTGACGATGAAGATC – 3’
P*yopH*-R	5’ – GCTTCCCTCCTTAATTAAATTAAG – 3’
**qRT-PCR**	
yspP-F	5’ – ACATCGCCCCAACACCTG – 3’
yspP-R	5’ – TGTCCTGAAGTTAATCCCCGG – 3’
yopH-F	5’ – GTTGGAGGCTGCATTGCG – 3’
yopH-R	5’ – TGCGGTGGCTCTGACTTC – 3’
dnaK-F	5’ – CGAACCAACAGCTGCTGC – 3’
dnaK-R	5’ – TCAGGCGGCTATCGAAGTC – 3’
